# Adaptive multi-degree of freedom Brain Computer Interface using online feedback: Towards novel methods and metrics of mutual adaptation between humans and machines for BCI

**DOI:** 10.1371/journal.pone.0212620

**Published:** 2019-03-06

**Authors:** Chuong H. Nguyen, George K. Karavas, Panagiotis Artemiadis

**Affiliations:** School for Engineering of Matter, Transport and Energy, Arizona State University, Tempe, Arizona, United States of America; Shanghai Jiao Tong University, CHINA

## Abstract

This paper proposes a novel adaptive online-feedback methodology for Brain Computer Interfaces (BCI). The method uses ElectroEncephaloGraphic (EEG) signals and combines motor with speech imagery to allow for tasks that involve multiple degrees of freedom (DoF). The main approach utilizes the covariance matrix descriptor as feature, and the Relevance Vector Machines (RVM) classifier. The novel contributions include, (1) a new method to select representative data to update the RVM model, and (2) an online classifier which is an adaptively-weighted mixture of RVM models to account for the users’ exploration and exploitation processes during the learning phase. Instead of evaluating the subjects’ performance solely based on the conventional metric of accuracy, we analyze their skill’s improvement based on 3 other criteria, namely the confusion matrix’s quality, the separability of the data, and their instability. After collecting calibration data for 8 minutes in the first run, 8 participants were able to control the system while receiving visual feedback in the subsequent runs. We observed significant improvement in all subjects, including two of them who fell into the BCI illiteracy category. Our proposed BCI system complements the existing approaches in several aspects. First, the co-adaptation paradigm not only adapts the classifiers, but also allows the users to actively discover their own way to use the BCI through their exploration and exploitation processes. Furthermore, the auto-calibrating system can be used immediately with a minimal calibration time. Finally, this is the first work to combine motor and speech imagery in an online feedback experiment to provide multiple DoF for BCI control applications.

## Introduction

In an effort to ameliorate rehabilitation and neural pathology treatment, Brain Computer Interfaces (BCI) aim to provide a solution where users can use brain signals to directly interact with the environment. Originally developed for patients with severe paralysis, a majority of research in BCI focuses on deciphering motor imagery to control external devices, such as a wheelchair [1]. Despite being successful at a considerable number of subjects [2, 3], BCI based on motor imagery still suffers from several deficiencies, which restrict its use in some practical applications. First, the conventional BCI systems often require a lengthy, off-line calibration step, which includes recording brain signals without feedback and training a statistic model, before it can be used. Second, BCI illiteracy is a well-known phenomenon observed in a non-negligible group of subjects, estimated at 15% to 30% [4], who are unable to generate modulation of sensorimotor rhythms detectable by current methods [3, 5]. Third, BCI systems can usually offer an only limited number of DoF. For instance, most BCI systems rely on binary classification, such as left vs. right hand imagery, whereas the highest number of DoF is achieved based on classification between four classes [6].

Recently, a new trend in BCI systems investigates the applicability of using speech imagery for control applications. Preliminary results reported by [7–14] are encouraging. Moreover, speech imagery also opens up the possibility of silent communication where sound recognition is prohibited, such as in noisy surroundings or in the case of locked-in patients with speaking disabilities. Compared to motor imagery, speech imagery is more natural, easier to perform repeatedly, and more consistent across users, since humans often unintentionally do it in daily activities, such as when reading a book silently, or during self-talking. Furthermore, speech can include arbitrary instructions, thus a user can associate a meaningful word to a corresponding action, which makes the interaction with the environment more intuitive.

Our primary aim in this work is alleviating the aforementioned drawbacks of current BCI systems by proposing an adaptive, online-feedback methodology based on the combination of motor and speech imagery tasks.

## Related work

Adaptive online learning BCI systems have been proved to be more effective than the conventional approaches. In adaptive online learning BCI, the classifier makes decisions and provides continuous feedback to the users while periodically updating its model and parameters. The adaptation techniques vary with respect to different aspects, i.e. the components that are being adapted (adaptive features vs adaptive classifier), the type of training (supervised vs semi or unsupervised learning), or at the user level, e.g. whether features are designed depending on subjects. In practice, adaptive BCI systems are often implemented based on a combination of different techniques.

Spuler et. al. [15] implemented an unsupervised, adaptive Support Vector Machine (SVM) classifier [16] to deal with the shift in the data covariance. In [17], the same authors proposed using PCA to improve the non-stationary effect in data during session transfer. Vidaurre et. al. [18, 19] proposed a system utilizing adaptive autoregressive (AAR) model to extract features and a quadratic discriminant analysis as a classifier. In [20], the authors investigated a combination of different features, such as AAR model parameters and Logarithmic Bandpower or their concatenation, and different classifiers, namely adaptive information matrix and Kalman adaptive LDA. In [5, 21], Vidaurre et. al. proposed a training paradigm comprising 3 adaptation levels, progressing from simple Laplacian channels based features, that are subject-independent in level 1, toward more complex ones, which included Laplacian channels, frequency bands and Common Spatial Patterns (CSP) designed specifically for each subject in level 3. The LDA classifier in that work was also designed adaptively from supervised in level 1 and 2 toward unsupervised in level 3 to deal with data-drifting between experimental sections [22]. Faller et. al. [23, 24] proposed a BCI system that selects the most discriminating frequency band using Fisher criteria, and retrains an LDA classifier after every 5 trials. Scherer et. al. [25] implemented a similar strategy, while also performed a calibration step for each user to select the 2 most discriminative among 4 tasks, such as subtraction, word association, hand or feet motion imagery. Positive results from the mentioned work demonstrated that online adaptive BCI learning is much more effective than the conventional offline, non-feedback systems. Especially, the methods can be potentially applied to subjects with BCI illiteracy or severe impairment [18].

However, the mentioned approaches were validated almost exclusively during binary classification of motor imagery tasks. More importantly, most of the adaptive systems focused on improving the machine learning component, while only a few systems ([5, 21, 24, 26, 27]) took into consideration the users’ adaptation counterpart.

First, we need to acknowledge that co-adaptation can introduce positive but also negative effects if not performed properly. If the classifier is correctly modified, the users will learn the system faster as they won’t need to change their mental processes as much. On the contrary, an inappropriate adaptation process can create the feeling of using a different system each time the classifier adapts. In that case, the user will need to change his/her mental processes significantly and more frequently. This not only causes confusion but also discouragement and frustration, which significantly impede the learning process.

Another fact that might have been overlooked by other adaptive systems is that, during the process of learning how to use the system, the subject needs to explore and exploit different ways to perform the mental tasks, while he/she may be also distracted by irrelevant thoughts (internal noise). Hence, the features that we can extract from the EEG signals may be scattered in the feature space, which at best can be represented by a mixture of Gaussian distributions. Since the data can drift back and forth during the exploration and exploitation process, retraining a classifier and using a single model based on the most recent data may not be an effective way to encourage the user adaptation, since older data/models might prove more effective.

Unfortunately, no metric can indicate with absolute precision whether the updated model converges or diverges from the user’s intent. In [28], Lotte et. al. pointed out that the classification accuracy, a measurement that is often used in the literature, is a poor metric to evaluate the user performance in online BCI training. Obviously, an enhancement in the prediction accuracy could merely be attributed to better tuning parameters for the current data and not necessarily to user improvement. Accordingly, a performance decrease could mainly occur because the user does not perform well in that particular experiment for reasons unrelated to the classifier, such as loss of focus or fatigue. Hence, co-adaptive systems must decouple the performance of the user from that of the classifier to evaluate the improvement of each component and their convergence.

Another ad-hoc problem for online-feedback adaptive classifiers is to decide the adaptive rate. Adaptive algorithms can be divided into two main approaches: sample-based and batch-based approaches. In the first method, such as [5, 18–21], the classifier modifies its parameters after receiving each new data sample. The general form of this approach is *θ*(*t* + 1) = *θ*(*t*) + *γe*(*t*), where *θ* is the classifier’s hyper-parameter, *γ* is the adaptive rate and *e*(*t*) is the error. Tuning *γ* is critical since each user performs differently. A small *γ* leads to slow and ineffective adaptation, while a large *γ* might lead to an unstable algorithm. This is also theoretically proved from the mathematic model of the two-learners problems proposed by Muller et. al. [29].

In batch-based approaches, such as [23–25], a new classifier is often retrained after a certain period of time or when the prediction fails below a certain accuracy level. The new classifier is often trained based on the new batch of data collected, but can reuse a portion of the previous data batch. When to retrain and what portion of the recent and previous data are used for retraining the classifier decide the adaptive rate in this case. Since EEG data are non-stationary and shift overtime, including too many samples from old data will introduce outliers to the training dataset, while using only new data can lead to an abrupt change in the classifier parameters. Furthermore, if the classifier is retrained after observing a decrease in the prediction accuracy, the new data may not be very discriminative to improve the model. In both methods, selecting representative data during the online learning to update the classifier is still an open question.

Regarding speech imagery, in our previous work [30], we conducted a literature review and investigated the applicability of different types of speech imagery for control applications. The main approach in [30] is based on a spatial covariance matrix (COV) descriptor and a Relevance Vector Machines (RVM) classifier. The COV descriptor has been widely used in computer vision [31–33], and recently adopted in BCI research as an effective feature for motor imagery classification [34–39]. Wang et. al. [40] did an investigation to combine motor and speech imagery to improve the DOF for BCI. However, their work was conducted offline without feedback. Moreover, they only investigated binary classication, either between two speech imagery tasks or between 1 motor imagery and 1 speech imagery task. Hence, it still cannot improve the number of DOF for a BCI system.

In this paper, we extend our previous work developed in [30] by proposing a multi-class, adaptive online-feedback BCI training paradigm toward the following objectives:

- Provide a simple but robust method to select data for updating the classifier.
- Propose an adaptive online-feedback methodology to improve the user learning experience by encouraging their exploration and exploitation process.
- Combine different modalities, e.g motor imagery and speech imagery, to perform control of multiple DoF.

## Materials and methods

### Experiment protocol

#### Main procedure

Eight healthy subjects (S1-8, 6 males and 2 females, ages 22-32) performed four mental tasks, namely two motor imageries of moving *left hand* (class 1) and *right hand* (class 3), and two speech imageries of saying a *long word* (class 2) and a *short word* (class 4). All subjects were right-handed except subject S3. S1 and S4 had experience in both off-line motor and speech imagery. S5, S6 and S8 had experience in off-line speech imagery, and the other subject participated in an EEG experiment for the first time. The experiment was approved by the ASU IRB (Protocols: 1309009601, STUDY00001345) and each participant signed an informed consent form before the experiment. The subjects were sitting in front of a computer monitor in a quiet and dark room. They were instructed to relax and keep both hands still for 5 minutes or until their hands felt numb before the experiment started. For motor imagery, the subjects were asked to imagine the kinesthetic sensation of closing and opening their hands without performing any actual motion. For speech imagery, they were instructed to pronounce a short word or long word internally in their minds and avoid any overt vocalization or muscle movements.

**Fig 1:**
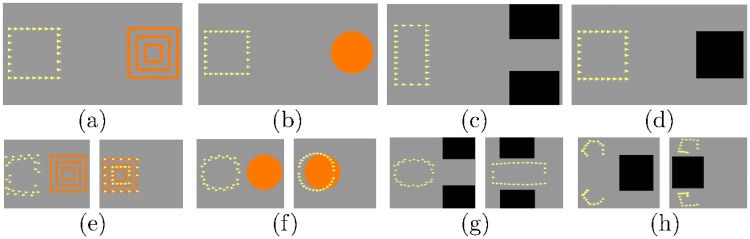
Simulation of swarm motion controlled by the proposed BCI system. Figs (a) and (b) assign the task of imagining moving the left hand (class 1) and the right hand (class 3) to control the swarm density and the shape of the formation, respectively. Figs (c) and (d) assign the task of imagining saying the words “concentrate” (class 2) and “split” (class 4) to concentrate and split the swarm, respectively. Fig (e,f,g,h) show the system’s feedback to the corresponding imagery of the users.

Inspired by our previous work [41], we associated the mental tasks with commands to control a swarm of robots’ behavior in simulation. Specifically, at the beginning of each trial, the swarm of robots is represented by yellow particles in a rectangular formation shown on the left of the screen, while a target is shown on the right. If the target is represented by orange concentric squares as shown in Fig 1a, the required mental task is to imagine moving the *left hand* to increase the *swarm density* (class 1). When the target is presented as an orange disk as shown in Fig 1b, the subject needs to imagine moving his/her *right hand* to control the *shape* of the swarm (class 3). When the target is displayed as two black squares as shown in Fig 1c, the subject needs to imagine saying the long word “concentrate” to *concentrate* the swarm toward the center and to pass it through (class 2). Finally, if the target is a single black square as shown in Fig 1d, the subject needs to imagine saying the short word “split” to *split* the swarm and avoid the obstacle (class 4). Subjects were asked to look only at the swarm to avoid any eye motion during the experiment.

Each experiment was conducted in a single day and approximately lasted for 2 hours, which included 30 minutes for preparation and 90 minutes for the main procedure. The main procedure had a total of 7 runs with 2−3 minutes break between them or until the subject felt ready for the next one. A single run was comprised of 40 trials, 10 for each class, which were shown randomly. There were random 2s or 3s pause between two consecutive trials, and a 10s break after the 15^th^ and 30^th^ trials in each run. The trial duration was 10s, and during the first 2s, the swarm and the target stayed still on the left and the right of the screen respectively, as illustrated in Fig 1(a), 1(b), 1(c) and 1(d). In this first 2s, the subject was also preparing to perform the corresponding imagination task. After that, the target moved from the right to the left, while the swarm maintained its center’s initial position but changed its formation according to the classifier prediction, as shown in the left of Fig 1(e), 1(f), 1(g) and 1(h). This motion simulation was intended to reduce user’s eye motion given the mentioned visual feedback. The classifier updated the prediction every 0.25*s*, hence a trial was completed after 33 steps, as shown in the right of Fig 1(e), 1(f), 1(g) and 1(h).

The first run (run 0) was used to collect data for training the initial model, thus we simulated the prediction of the classifier by randomly showing the correct (expected) motion of the swarms with 80% probability and an unexpected random motion with 20% probability. The purpose was to help the subjects get used to the distraction of the prediction’s inaccuracy. The subjects were aware of this, but were asked to treat it as true feedback. In run 1 to 4, the swarm’s motion was updated only when the classifier predicted correctly, otherwise it stayed still. Finally, in the last two runs, the classifier updated the swarm motion without the aforementioned constraint. This incremental change of the challenge level was chosen in order to help the user be more concentrated and confident during the adaptation, while also help us investigate different levels of the exploration and exploitation processes.

#### Data acquisition and conditioning

The EEG signals were acquired using a BrainProducts ActiCHamp amplifier system from 64 electrodes placed according to the 10/20 International system [42]. Among them, 60 channels were used to extract the features, while 4 other were used to keep track of the ElectroOculoGraphic (EOG) components [43]. The data were recorded at 1000Hz and then downsampled to 256Hz for processing. A 5^th^ order Butterworth bandpass filter between 8-70 Hz was applied to remove any low-frequency trends in the data as well as possible artifacts related to ElectroMyoGraphic (EMG) activity. This frequency band is selected as we found it is efficient for speech imagery [30]. A notch filter at 60Hz was also applied in order to remove line noise. Finally, an EOG artifact removal algorithm [43] was applied on the data to eliminate any eye blinking or eye movement artifacts.

### Preliminary

Following the standard notations, we denote ℜ^*n*^ as an *n* dimension real space, ***I***_*n*_ ∈ ℜ^*n*×*n*^ the identity matrix and ***A***^T^ the transpose of ***A***. diag(***x***) is the diagonal matrix constructed from ***x***, and λ_*i*_ = eig_i_(A) is the *i*^th^ eigenvalue of ***A***. ∥ ⋅ ∥ denotes the vector Euclidean norm or matrix Frobenius norm.

#### Common Spatial Pattern (CSP)

CSP [44–47] is an effective method to extract discriminative channels for the mental tasks. CSP seeks for the linear transform ***W*** mapping the data collected from *N* channels to a space of *n* < *N* useful channels, ***Y*** = ***W***^T^ ***X***. Grosse-Wentrup [48] combined Mutual Information and Joint Approximate Diagonalization to generalize CSP for multi-class applications. In this work, we will apply this approach [48] for spatial filter as it is suitable for multiple tasks.

#### Distance on Riemannian Manifold

**Definition 0.1** *An n* × *n matrix* ***A*** *is Symmetric Positive Definite (SPD) if* ***A*** = ***A***^T^, ***x***^T^ ***Ax*** > 0, ∀***x*** ≠ 0. *Equivalently*, λ(***A***) > 0. *A SPD matrix is considered as a point on the Riemannian Manifold denoted by* ${\text{Sym}}_{\text{n}}^{\text{+}}$ [49].

**Definition 0.2** *Let X* ∈ ℜ^*n*×*T*^ *be the EEG signals of n channels and T time samples*, *the Spatial Covariance Matrix (COV) descriptor is defined as* $C=\frac{XX^{\text{T}}}{T-1}\in {\text{Sym}}_{\text{n}}^{\text{+}}$.

**Definition 0.3** ***A***^*k*^, exp(***A***) *and* log(***A***) *of a SPD matrix* ***A*** ∈ ℜ^*n*×*n*^ *are defined through its eigenvalues* **Λ** *and eigenvectors **U*** as [49]:

$$\begin{array}{cc} A^{k} & ≜U\;\text{diag}\left(\left[\lambda_{1}^{k},\cdots ,\lambda_{n}^{k}\right]\right)U^{T}=U\Lambda^{k}U^{T},\\ \text{exp}(A) & ≜U\;\text{diag}\left(\left[e^{\lambda_{1}},\cdots ,e^{\lambda_{n}}\right]\right)U^{T}=Ue^{\Lambda }U^{T},\\ \text{log}(A) & ≜U\;\text{diag}\left(\left[\text{log}\left(\lambda_{1}\right),\cdots ,\text{log}\left(\lambda_{n}\right)\right]\right)U^{T}=U\;\text{log}\left(\Lambda \right)U^{T}. \end{array}$$

Since SPD matrices are in the Riemannian Manifold, Riemanian distance is more effective than Euclidean distance to discriminate them. In [50], a detailed description and a comparison of the performance between different metrics on ${\text{Sym}}_{\text{n}}^{\text{+}}$ is conducted in the context of BCI applications. In this work, we use two distance metrics:

1. Riemannian Distance [51] between ***S***_1_ and ***S***_2_ which is
  $$\begin{array}{c} d_{R}^{2}\left(S_{1},S_{2}\right)≜{∥\text{log}\left(S_{1}^{-1}S_{2}\right)∥}^{2}=\sum_{i=1}^{n}{\left(\text{log}\left(\lambda_{i}\right)\right)}^{2}, \end{array}$$ (1)
  where $\lambda_{i}={\text{eig}}_{i}(S_{1}^{-1}S_{2})$. This metric is invariant to affine transforms and inversion. However, it is computationally expensive, and often approximated by the Euclidean distance between their tangent vectors.
2. Euclidean Distance between Tangent Vectors
  - *The tangent vector* ***T*** of a point ***S*** at the reference point ***C*** is defined as
    $$\begin{array}{c} T={\text{log}}_{C}\;S≜\text{log}\left(C^{-\frac{1}{2}}SC^{-\frac{1}{2}}\right). \end{array}$$ (2)
  - The distance between ***S***_1_ and ***S***_2_ on the Riemannian manifold can be derived through the Euclidean distance between the tangent vectors as
    $$\begin{array}{c} d_{TS}^{2}{\left(S_{1},S_{2}\right)}_{C}≜{∥T_{1}-T_{2}∥}^{2}. \end{array}$$ (3)

The reference point ***C*** can be selected as ***I***_*n*_, or the geometric mean of the dataset. For better accuracy, the geometric mean is often used, and this process is called normalization. In this work, we use the geometric Karcher mean [52], which can be obtained by an iteration algorithm described in Algorithm 1 [53, 54].

**Algorithm 1** Riemannian Mean of Covariance Matrices

**Input**: Training dataset ${\left\{S_{i}\right\}}_{i=1}^{n}\in {\text{Sym}}_{\text{m}}^{\text{+}}$, and tolerance *ϵ*_b_ > 0

**Output**: Mean $\bar{S}$ of $\{S_{i}{\}}_{i=1}^{n}$.

**Initialize**: Mean $\bar{S_{0}}=\frac{1}{n}\sum S_{i}$.

**Procedure**: For *k* = 0…*n*_iter_, do:

 *1*. *For i* = 1…*n*, *find tangent vector*: $T_{i}={\text{log}}_{{\bar{S}}_{k}}(S_{i})$.

 *2*. *Find the Euclidean mean*: $\bar{T_{k}}=\frac{1}{n}\sum_{i=1}^{n}T_{i}$.

 *3*. *Map* ${\bar{T}}_{k}$ *back to* ${\text{Sym}}_{\text{m}}^{\text{+}}$: ${\bar{S}}_{k+1}={\bar{S}}_{k}^{\frac{1}{2}}\text{exp}({\bar{T}}_{k}){\bar{S}}_{k}^{\frac{1}{2}}$.

 *3*. *If* ∥***T***_*k*_ − ***T***_*k*−1_∥ < *ϵ*_b_, *break*.

#### Relevance Vector Machine classifier

RVM [55] is an extension of the more popular Support Vector Machines (SVM) classifier. Different from SVM, RVM has the following advantages:

- RVM is a native multiple-class Bayesian Classifier, and its prediction output is the probabilistic confidence of a sample belonging to different classes.
- RVM assumes that the whole dataset can be represented by sparse representative data points. To construct the decision boundary, the weights of these data points, i.e. the relevance vectors, are optimized automatically based on the Bayesian principle. Thus RVM avoids the over-fitting problem without the tuning requirement for the hyper-parameters, such as the cross-validation in SVM.
- RVM is a sparse classifier. Thus, RVM can predict data more efficiently and faster than SVM.

More details in comparing RVM and SVM usage in BCI can be found in our previous work [30, 50]. In this paper, we use the multi class RVM (mRVM) proposed by [56, 57].

### Proposed method

#### Spatial filter and data selection for training

If the COV is computed from all channels, the feature vector not only contains noise but is also high dimensional, thus computationally expensive to process. Hence, selecting relevant channels is critical to improve the accuracy and efficiency. This is done by applying an appropriate filter using the CSP methodology described further below.

Furthermore, since the classifier predicts the mental tasks every 0.25s, a 10s trial yields total *N*_seg_ = 33 segments of 2s duration with 1.75s overlap. All segments in a trial have the same label, which is the task assigned at the beginning of each trial as illustrated in Fig 1. Hence, from each run, we obtain 330 data points (10 trials x 33 segments) for each class or 1320 labeled samples in total.

However, not all datapoints are useful to train the model, thus selecting representative ones is necessary to reduce the noise and improve the training speed. Because the users were asked to repeat each mental task several times until the end of a trial, e.g. imaging of saying “split” at the same way and the same rhythm, each trial is expected to contain repetitions of a central unique pattern. Thus, from 33 data points in one trial, we can select representative data as the *k*-nearest neighbors (*k*-NN) of their Riemannian mean. Parameter *k* is important, as selecting a few will not capture the diversity, while too many will include noisy data. Hence, *k* is chosen by a cross-validation procedure described shortly.

Let {***X***_*i*,*j*_} be the dataset collected in one run, where ***X***_*i*,*j*_ ∈ ℜ^*D*×*T*^ is the 2s segment data with *D* = 60 channels and *T* = 512 being the number of datapoints (2s at 256Hz); *i* = 1: *N*_seg_ is the segment index, and *j* = 1: *N*_trial_ is the trial index, $\hat{y}(X_{ij})$ and *y*_*j*_ are the predicted and the true label of sample *X*_*i*,*j*_. We propose Algorithm 2 to simultaneously compute the spatial filter and select representative data for training the classifiers.

**Algorithm 2** Spatial Filter and Sample Selection for Training

**Input**: Dataset {***X***_*i*,*j*_} and number of CSP channels *d* < *D*.

**Output**: CSP matrix ***W*** ∈ ℜ^*D*×*d*^ and the set *T* = {*T*_*j*_}, where *T*_*j*_ = {*i*} is the index set of segments ***X***_*i*,*j*_ chosen from trial *j*.

**Initialize**: ***W*** by computing the CSP matrix using all segment {***X***_*i*,*j*_} for run 0. Otherwise, ***W*** is the CSP matrix from the previous run.

**Pre-filter**: Select samples that have: $P(\hat{y}(X_{ij})=y_{j})>\epsilon_{\text{f}}$.

**Procedure**:

 *1*. *Apply spatial filter on the data*: ***Z***_*i*,*j*_ = ***W***^T^ ***X***_*i*,*j*_ and $C_{ij}=\frac{Z_{i,j}Z_{i,j}^{\text{T}}}{T-1}$.

 *2*. *For each trial j*

  • Find the mean *μ*_*j*_ of ${\left\{C_{ij}\right\}}_{i=1:N_{\text{seg}}}$ using Algorithm 1.

  • Select *T*_*j*_ = {*i*}, s.t. ***C***_*ij*_ is a *k*-NN of *μ*_*j*_ based on (1).

 *3*. *Compute new* ***W*** as a CSP matrix of the selected index *T* = {*T*_*j*_} using the multi-class CSP algorithm [48].

In Algorithm 2, the Pre-filter step can be applied, where $P(\hat{y}(X_{ij})=y_{j})$ is the probability predicted by the previous model during the online testing. We can choose *ϵ*_f_ small, e.g. *ϵ*_f_ = 0.1 < *P*_chance_ = 0.25, to reject certainly noisy samples, but keep the data selection of a new run as independent to the models build from previous runs as possible to accommodate the user exploration process.

#### Training of a Relevance Vector Machine model

**Fig 2:**
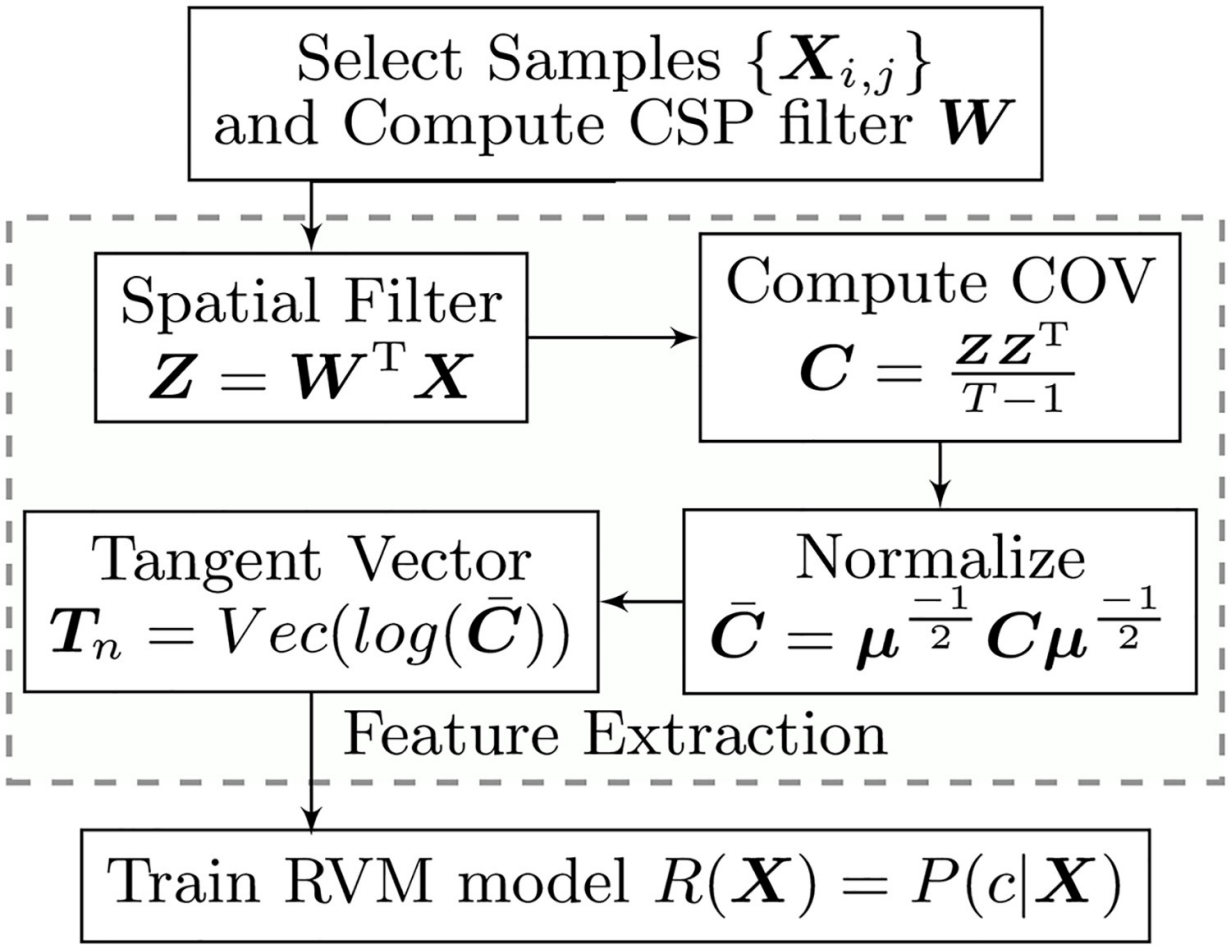
Training Procedure for a sub-model Relevance Vector Machine.

Fig 2 summarizes the steps of training each RVM model.

1. Step 1: We apply Algorithm 2 on the raw dataset from the most recent run. This yields a subset of representative data {***X***_*i*,*j*_} and a CSP matrix ***W*** ∈ ℜ^*D*×*d*^.
2. Step 2: We extract the tangent vector as features. First, we apply the spatial filter and compute the COV matrix $C\in {\text{Sym}}_{\text{+}}^{\text{d}}$. Then, we compute the mean ***μ*** of the dataset {*C*_*ij*_} using Algorithm 1, and use ***μ*** as the reference point to normalize this dataset. The final feature vector ***T***_*n*_ is obtained by vectorizing the upper half of matrix $log(\bar{C})$ and scale the off-diagonal elements by $\sqrt{2}$.
3. Step 3: We train the RVM model [56, 57] using the distance metrics (3). The model can predict the probability *P*(*c*|***X***) of a sample ***X*** belonging to a class *c*.

#### Mixture of RVM models

**Fig 3:**
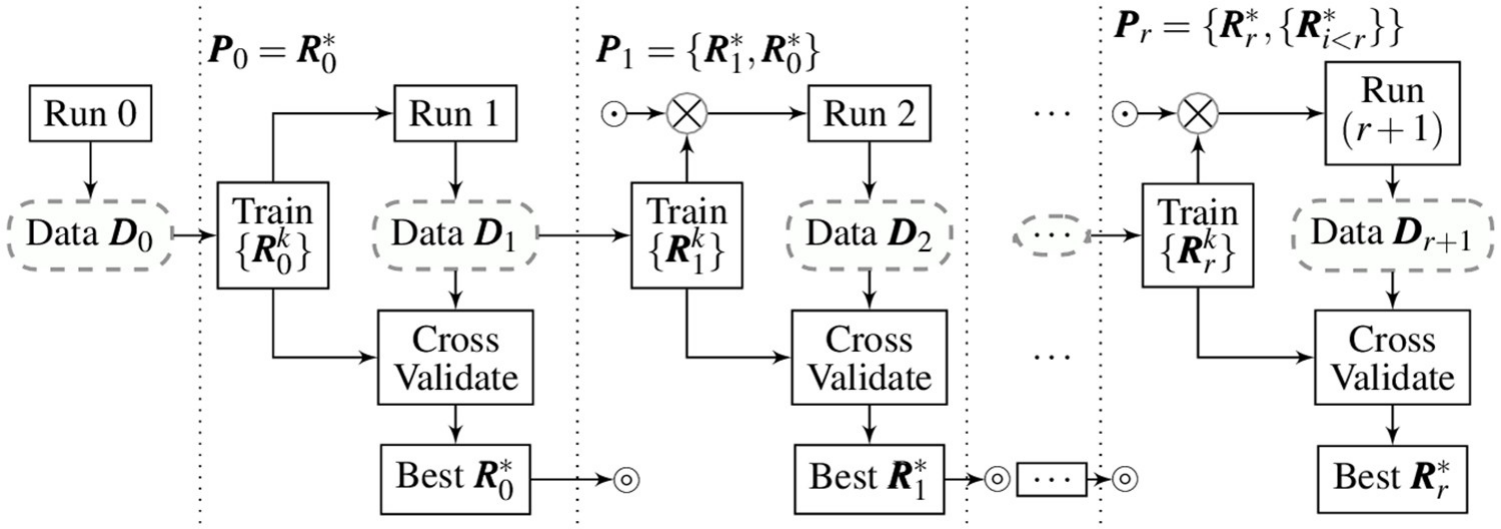
Procedure of the proposed online learning mixture of RVM models. Symbols ⊚ and ⊙ represent the In and Out connection respectively.

Fig 3 illustrates the process of updating the proposed mixture of RVM classifiers. Concretely, we collect a dataset ***D***_*r*_ = {***X***_*i*,*j*_} after each run *r* to train a set of RVM models $\left\{R_{r}^{k}\right\}$, each of which corresponds to a selection of *k* = 8, ‥, 12 data points. The model $R_{r}^{*}(X)$ with *k* = k* is selected to combine with other optimal models $\left\{R_{t}^{*},t<r\right\}$ obtained previously to form the mixture models:

$$\begin{array}{c} P_{r}\left(X\right)=R_{R}^{*}\left(X\right)\cup R_{R-1}^{*}\left(X\right)\cup \left\{R_{t<R-1}^{*}\left(X\right)\right\} \end{array}$$ (4)

The number of data points *k* is also an important factor to balance between the noise and the user exploration process. After the run *r*, we perform two modifications to the mixture of models:

- We update the model $R_{r-1}^{*}$. To select the optimal model $R_{r-1}^{*}$ from the set $\left\{R_{r-1}^{k}\right\}$, the dataset ***D***_*r*_ is used for cross-validation. We rank the performance of $R_{r-1}^{k}$ based on the quality *p*(***Q***) of the confusion matrix ***Q*** tested on ***D***_*r*_:
  $$p\left(Q\right)=\sqrt{\text{min}(q)\text{mean}(q)}$$ (5)
  where ***q*** is the diagonal of ***Q***. Note that, in contrast to the average accuracy, i.e. mean(***q***), the quality *p*(***Q***) emphasizes on the performance balance between the classes, s.t. *p*(***Q***) is maximized if min(***q***) = mean(***q***), or the accuracies for all classes are equal.
- We add the new trained model $R_{r}^{*}$ by inferring. Here, due to absence of ***D***_*r*+1_, we can not cross-validate the model set $\left\{R_{r}^{k}\right\}$ prior to the run *r* + 1. Hence, the optimal index k* obtained from the cross-validation on the set $\left\{R_{r-1}^{k}\right\}$ is used to infer the optimal model of the set $\left\{R_{r}^{k}\right\}$. In run 1, we heuristically select the model $\left\{R_{0}^{k^{*}=9}\right\}$ as k* = 9 often yields satisfied results in our preliminary study.

The mixture of RVM models is then defined as

$$P_{r}\left(X_{t}\right)=\sum_{i=r+1-m}^{r}w_{i}\left(t\right)R_{i}^{*}\left(X_{t}\right),$$ (6)

where *m* is the number of sub-models, and *w*_*i*_(*t*) is the time-dependent weight of each sub-model $R_{i}^{*}(X_{t})$. The next section will discuss how to update the weight *w*_*i*_(*t*) online.

### Online adaptive mixture of RVM models

**Fig 4:**
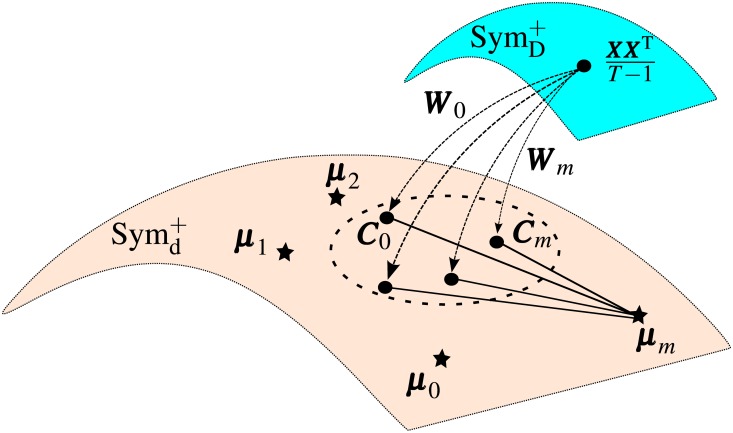
Estimate weights *w*_*i*_ for the mixture of RVM models.

Since each sub-classifier ***R***_*i*_(***X***) is equipped with a spatial filter ***W***_*i*_ and a mean $\mu_{i}\in {\text{Sym}}_{\text{d}}^{\text{+}}$, a test sample ***X***_*t*_ is mapped to *m* points $C_{i}=W_{i}^{\text{T}}\frac{XX^{\text{T}}}{T-1}W_{i}\in {\text{Sym}}_{\text{d}}^{\text{+}}$ as illustrated in Fig 4.

Each ***C***_*i*_ is then fed to each sub-model ***R***_*i*_(*X*) to compute their response. Moreover, *d*(***C***_*i*_, ***μ***_*j*_), the Riemannian distance from ***C***_*i*_ to the mean ***μ***_*j*_, also reflects how similar the sample to the dataset ***D***_*j*_ is, thus how suitable to use the sub-model ***R***_*j*_(***X***) to predict the sample. Hence, we define the weight *w*(*t*) in (6) as

$$w_{i}\left(t\right)=\frac{b_{i}\left(t\right)}{\sum_{j=r+1-m}^{r}b_{j}\left(t\right)},\;\;b_{i}\left(t\right)=\frac{a_{i}}{{\bar{d}}_{i}\left(t\right)}$$ (7)

where ${\bar{d}}_{j}$ is the mean of the set *d*(***C***_*i*_, ***μ***_*j*_).

In (7), *a*_*i*_ can be used to specify the fixed prior of each model. Specifically, we set *a*_*i*_ = *p*(***Q***_*i*_) for the model $R_{i}^{*}(X),i<r$, where ***Q***_*i*_ is the confusion matrix of ***R***_*i*_ tested on ***D***_*r*_. *a*_*r*_ takes the maximum value of {*p*(***Q***_*i*_)}. The model *i* can be suppressed, i.e. set *a*_*i*_ = 0 if *p*(***Q***_*i*_) < *ϵ*, where *ϵ* (default *ϵ* = 0.25) is a threshold depending on the subjects.

In summary, our model utilizes two adaptation techniques.

- First, we update the components of the mixture of RVM models after each completed run to account for the data-shifting. In here, the feature adaptation is performed by recomputing the CSP and the COV reference matrix. This mini-batch adaptation approach also offers an adequate time for the user to adapt. Hence, during human adapting process, components of the mixture model are fixed. Thus, we interchangeably keep one part of the co-adaptive eco-system constant while the other adapts in order to safely prevent the potential diverge of the two systems if they adapt simultaneously.
- During the online test/feedback, the weight of each model changes between runs as described in (7). Hence, the mixtured models adapt to the user by incorporating the supervised knowledge collected previously through *a*_*i*_ and the user tendency during learning through ${\bar{d}}_{i}$.

## Results

### Evaluation

To evaluate the performance of the adaptive classifier, we compute the classification accuracy as a reference criterion since it is commonly used by other online BCI methods [5, 18–24, 26, 58]. The accuracy is computed for every segment in each trial, which yields a total of 1320 data points for each run. To make a conservative evaluation, we include all segments even though many of them can be safely removed, e.g. discard a segment if its maximum probability is less than 30%. These segments could be due to the user’s unintented moments, thus discarding them may further increase the reported accuracy.

We then show that a more proper and restrictive criterion is the quality of the confusion matrix. Note that, the chance level for four classes, i.e. always picking the same one choice among four, yields 25% accuracy, but 0 if we consider the quality of the confusion matrix. The confusion matrix quality is a more proper metric of the classifier’s efficiency because it takes into account not only the overall accuracy of the classifier but also its class-wise imbalance. In particular, it penalizes classifiers whose accuracy rate is extremely high for only a few particular classes, while extremely low for the others, thus biasing the overall accuracy and hiding their inefficency. At the same time, this criterion favors classifiers with high accuracy across all classes. Even if the overall accuracy is lower in these cases, the classifier with the highest quality value lead to a more balanced performance across all classes.

As mentioned, the accuracy and the confusion matrix quality cannot properly explain whether the classifier performance is improved due to user adaptation, or classifier adaptation or both. Hence, we further evaluate the performance of the user independently of the classifier based on two criteria, namely the separability and the instability of the data.

Separability between two classes A and B is defined as $s(A,B)=\frac{d(\mu_{A},\mu_{B})}{\sqrt{\sigma_{A}\sigma_{B}}},$ where *μ*_*i*_ is the Riemannian mean of class *i*, and *σ*_*i*_ is the standard deviation of the distances from all samples belonging to class *i* to *μ*_*i*_. Hence, a larger *s*(*A*, *B*) indicates that the two classes A and B are more separable. A slightly different criterion was also proposed in [28].

To evaluate the data instability, we first perform PCA on the tangent vectors of each class, where the Riemannian mean of each class is used as the reference point to normalize this class’s data. The tangent vector represents the direction of each point relative to the mean, thus, essentially capturing the directional distribution of dataset in the Riemannian space. Hence, we denote the data’s instability as the number of principle components that can represent 95% of the data. The more components we choose, the higher variance and higher instability the data have.

To compute the separability and instability, we first remove any irrelevant channels by applying the multi-class CSP [48] with 12 CSP channels. Since each component of the RVM mixture model is equipped with a CSP matrix obtained from the data in the previous run, we can re-apply the CSP matrix obtained from the dataset *D*_*r*−1_ to *D*_*r*_ (Method 1). In this way, we can reconstruct the separability and instability of data during the experiment. Another way is to compute a new CSP matrix using *D*_*r*_ for run *r* (Method 2). Note that, this CSP matrix can only be obtained after completing the run *r*, hence it is not available during the online testing. To evaluate the user performance independently of the classifiers, we prefer the second method.

### Classification results

Tables 1 and 2 report the prediction accuracy and the quality of confusion matrix (QCM) respectively.

**Table 1:** Prediction accuracy at each run *D*_*i*_.

	Constrained Feedback				Full feedback	
Sub	*D*_1_	*D*_2_	*D*_3_	*D*_4_	*D*_5_	*D*_6_
S1	42.5	46.4	49.7	49.6	58.9	55.8
S2	37.5	45.5	54.2	50.1	47.6	52.1
S3	31.8	32.3	32.0	34.8	32.9	36.8
S4	52.0	59.0	58.5	55.1	54.4	54.6
S5	68.6	70.6	73.4	65.8	61.5	61.9
S6	34.1	34.0	42.5	48.3	33.0	58.1
S7	48.4	52.2	53.3	64.9	51.2	63.6
S8	34.7	30.6	43.7	42.6	36.9	30.7
**Avg**	44.1	46.4	51.6	51.0	47.1	52.5

**Table 2:** Quality of Confusion Matrix (QCM) at each run *D*_*i*_.

	Constrained Feedback				Full feedback	
Sub	*D*_1_	*D*_2_	*D*_3_	*D*_4_	*D*_5_	*D*_6_
S1	29.6	39.0	41.3	41.4	52.8	36.5
S2	30.5	33.6	45.8	47.1	41.1	44.1
S3	18.4	18.7	32.3	27.9	16.5	19.5
S4	41.8	50.0	50.2	48.0	40.6	31.5
S5	56.4	54.1	67.0	45.5	44.9	41.3
S6	21.5	27.4	35.7	42.8	21.5	50.7
S7	31.1	21.4	37.9	63.8	46.4	42.8
S8	10.2	14.3	27.2	36.1	13.4	11.4
**Avg**	29.9	32.3	42.2	44.1	34.6	34.7

**Fig 5:**
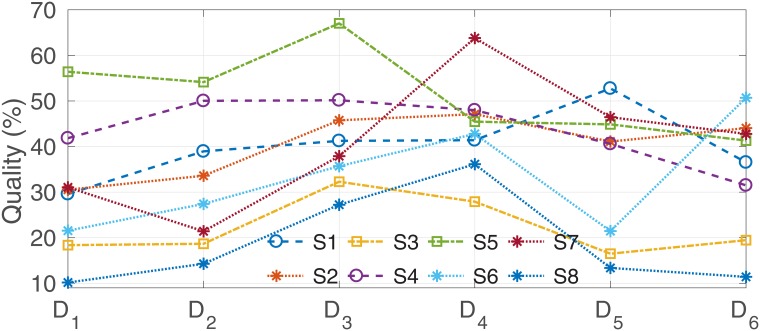
Confusion matrix’s quality of subjects S1-S8 over runs *D*_1_ − *D*_6_.

Fig 5, which visualizes Table 2, shows that the classification results are improved after each run, and tend to reach the maximum at the run ***D***_4_, right before changing the feedback’s difficulty level. When the feedback in run ***D***_5_ became more aggressive by removing the constraints, the performance decreased as expected. As shown in last row of Table 2, we observed the averages of run ***D***_5_ and ***D***_6_ decreased about 10% relative to run ***D***_3_ and ***D***_4_. However, in run ***D***_6_, subjects S2, S3, S6 were able to regain control, as the quality increased relatively to run ***D***_5_. For subjects S5, S7, S8, we observed a slight decrease, approximately 3%.

To show that QCM is more proper to evaluate the classification performance than accuracy, we can look at several particular data pairs colored in both Tables 1 and 2. In these data pairs, although the accuracy slightly changed (< 4%), their QCM could reduce (orange color) or increase (blue color) significantly (> 10%). This is due to the bias in classification results, which the accuracy metric fails to capture.

To evaluate if our proposed protocol can improve the performance significantly, we conducted the Wilcoxon left-tail signed rank test on the user performance given in Tables 1 and 2. The test results with 5% significant level are shown in Table 3, in which the first row are the p-values when compared between run ***D***_*i*−1_ and run ***D***_*i*_, and the second row is between ***D***_1_ and ***D***_*i*_. Bold number indicates that the improvement was statistically significant (< 5%), and indeed appeared in run ***D***_3_ and ***D***_4_.

**Table 3:** P-value of Wilcoxon left-tail signed rank test on performance using Accuracy (Table 1) and QMC (Table 2).

	Accuracy					Quality of Confusion Matrix				
Run	*D*_2_	*D*_3_	*D*_4_	*D*_5_	*D*_6_	*D*_2_	*D*_3_	*D*_4_	*D*_5_	*D*_6_
***D***_*i*−1_	0.07	**0.02**	0.57	0.96	0.125	0.15	**0.004**	0.32	0.98	0.77
***D***_1_	0.07	**0.003**	**0.01**	0.08	0.055	0.15	**0.004**	**0.02**	0.23	0.19

To evaluate the improvement of each individual subject under the constrained feedback, we computed the slope *a* of the linear regression (*y* = *ar* + *b*) for QCM data (*y*) over run *r* = 1, …, 4. The results are shown in Table 4, which indicate that all but subject *S*_5_ had their performance improve over time (*a* > 0), and subject *S*_7_ had the highest improvement (*a* = 11.5). Subject *S*_5_ had a large step improvement in run ***D***_3_(12.9%), also the highest QCM (67.0) of all subjects, but then steeply decreased in run *D*_4_(−21.5%). Thus, although we observe the improvement of the subject *S*_5_ at the intermediate step during the experiment, the slope *a* is negative (also refer to Fig 5).

**Table 4:** Linear regression of QCM for each subject *S*_1−8_ over runs *D*_1−4_.

	*S*_1_	*S*_2_	*S*_3_	*S*_4_	*S*_5_	*S*_6_	*S*_7_	*S*_8_
*a*	3.77	6.19	4.22	1.86	−1.99	7.2	11.5	9.08
*b*	28.4	23.8	13.8	42.9	60.7	13.9	9.5	−0.8

### User performance via data’s separability

Tables 5–12 report the data separability score for each pair of classes for each subject, and the corresponding p-value of Wilcoxon left tail signed rank test. Table 13 shows that different types of imagery are more separable than similar ones, and ranks their separability in ascending order. Concretely, the separability of the speech imagery pair (2-4) and the motor imagery pair (1-3) are the lowest, while the ones for the pair (“Left hand”-“Split”) (1-4) and (“Right Hand”,“Split”)(3-4) are the most discriminable.

**Table 5:** Separability score of data for each class pair. (a)-(h) are the separability value of subject *S*_1_.

Pair	*D*_1_	*D*_2_	*D*_3_	*D*_4_	*D*_5_	*D*_6_
1 − 2	2.14	2.52	1.84	2.24	2.26	2.77
1 − 3	1.45	1.20	1.35	0.79	1.21	1.61
1 − 4	2.15	1.94	3.63	2.38	2.47	3.50
2 − 3	2.26	2.54	1.90	1.45	1.82	3.29
2 − 4	0.86	1.06	1.32	1.12	0.96	1.85
3 − 4	2.23	1.92	3.68	1.51	1.95	4.07
w.r.t ***D***_*i*−1_		0.50	0.28	0.97	0.08	**0.02**
w.r.t ***D***_1_		0.50	0.22	0.84	0.72	**0.02**

**Table 6:** Separability score of data for each class pair. (a)-(h) are the separability value of subject *S*_2_.

Pair	*D*_1_	*D*_2_	*D*_3_	*D*_4_	*D*_5_	*D*_6_
1 − 2	2.64	1.54	1.30	1.88	0.76	1.74
1 − 3	1.20	0.96	0.70	1.14	0.88	1.33
1 − 4	2.22	2.13	0.95	1.52	0.97	0.92
2 − 3	2.30	1.61	1.26	1.02	1.31	2.94
2 − 4	1.20	1.01	0.48	0.38	0.41	0.57
3 − 4	2.02	2.34	0.96	0.86	1.76	1.62
w.r.t ***D***_*i*−1_		0.92	1.00	0.22	0.66	0.08
w.r.t ***D***_1_		0.92	1.00	1.00	1.00	0.89

**Table 7:** Separability score of data for each class pair. (a)-(h) are the separability value of subject *S*_3_.

Pair	*D*_1_	*D*_2_	*D*_3_	*D*_4_	*D*_5_	*D*_6_
1 − 2	1.43	1.63	2.01	1.51	1.48	1.49
1 − 3	0.87	1.14	1.29	0.86	1.04	1.20
1 − 4	2.58	1.57	2.36	1.32	1.31	1.15
2 − 3	1.45	1.52	1.64	1.49	1.18	1.94
2 − 4	2.07	1.01	1.46	1.11	0.72	0.90
3 − 4	2.41	1.35	1.78	1.19	0.97	1.38
w.r.t ***D***_*i*−1_		0.84	**0.02**	1.00	0.95	0.08
w.r.t ***D***_1_		0.84	0.72	0.89	0.95	0.84

**Table 8:** Separability score of data for each class pair. (a)-(h) are the separability value of subject *S*_4_.

Pair	*D*_1_	*D*_2_	*D*_3_	*D*_4_	*D*_5_	*D*_6_
1 − 2	3.64	3.73	2.56	2.66	3.02	3.66
1 − 3	1.42	1.92	1.75	1.70	3.67	1.75
1 − 4	3.57	3.17	2.92	3.45	3.65	3.05
2 − 3	3.04	5.05	3.71	3.41	4.39	3.14
2 − 4	1.81	1.97	1.47	1.64	1.03	1.29
3 − 4	3.04	4.39	4.33	4.52	5.42	2.67
w.r.t ***D***_*i*−1_		0.08	1.00	0.22	0.08	0.92
w.r.t ***D***_1_		0.08	0.50	0.34	0.16	0.84

**Table 9:** Separability score of data for each class pair. (a)-(h) are the separability value of subject *S*_5_.

Pair	*D*_1_	*D*_2_	*D*_3_	*D*_4_	*D*_5_	*D*_6_
1 − 2	3.40	2.89	3.74	3.46	3.69	4.94
1 − 3	0.95	1.09	1.38	2.02	1.91	2.45
1 − 4	3.48	3.09	6.45	3.57	4.23	5.04
2 − 3	3.70	2.85	2.87	6.39	4.74	6.58
2 − 4	1.04	0.62	1.02	0.86	0.80	1.03
3 − 4	3.82	3.08	5.00	6.67	5.49	6.77
w.r.t ***D***_*i*−1_		0.98	**0.02**	0.34	0.78	**0.02**
w.r.t ***D***_1_		0.98	0.16	0.08	**0.03**	**0.03**

**Table 10:** Separability score of data for each class pair. (a)-(h) are the separability value of subject *S*_6_.

Pair	*D*_1_	*D*_2_	*D*_3_	*D*_4_	*D*_5_	*D*_6_
1 − 2	1.31	2.11	2.23	2.67	3.16	2.07
1 − 3	0.62	1.28	1.04	1.44	1.34	0.87
1 − 4	1.16	1.97	1.83	2.24	2.58	1.70
2 − 3	0.96	2.53	1.30	2.78	1.59	1.61
2 − 4	0.56	1.22	0.72	1.37	0.97	0.99
3 − 4	0.82	2.30	1.04	2.28	1.27	1.29
w.r.t ***D***_*i*−1_		**0.02**	0.98	**0.02**	0.84	0.84
w.r.t ***D***_1_		**0.02**	**0.02**	**0.02**	**0.02**	**0.02**

**Table 11:** Separability score of data for each class pair. (a)-(h) are the separability value of subject *S*_7_.

Pair	*D*_1_	*D*_2_	*D*_3_	*D*_4_	*D*_5_	*D*_6_
1 − 2	2.41	3.54	2.22	2.09	1.58	3.71
1 − 3	0.73	0.94	0.70	1.02	0.74	2.35
1 − 4	1.83	3.41	2.49	2.20	1.45	3.38
2 − 3	1.80	1.74	1.90	2.01	1.70	3.51
2 − 4	0.61	0.86	0.92	0.59	0.45	0.68
3 − 4	1.29	1.59	2.03	2.02	1.48	3.04
w.r.t ***D***_*i*−1_		**0.03**	0.78	0.78	1.00	**0.02**
w.r.t ***D***_1_		**0.03**	0.11	0.16	0.89	**0.02**

**Table 12:** Separability score of data for each class pair. (a)-(h) are the separability value of subject *S*_8_.

Pair	*D*_1_	*D*_2_	*D*_3_	*D*_4_	*D*_5_	*D*_6_
1 − 2	1.67	1.11	1.55	1.06	1.11	0.93
1 − 3	1.27	0.91	1.20	0.65	0.85	0.74
1 − 4	1.45	0.79	1.32	0.90	0.98	0.80
2 − 3	1.80	1.35	1.63	1.08	1.38	1.42
2 − 4	1.60	0.90	1.39	1.16	1.24	1.19
3 − 4	1.50	0.91	1.33	0.87	1.16	1.18
w.r.t ***D***_*i*−1_		1.00	**0.02**	1.00	**0.02**	0.95
w.r.t ***D***_1_		1.00	1.00	1.00	1.00	1.00

**Table 13:** Range of separability score for each mental task pair taken from all subject’s runs.

Pair	Min	Median	Max	Rank
1 − 2	2.14	2.52	1.84	3
1 − 3	1.45	1.20	1.35	2
1 − 4	2.15	1.94	3.63	5
2 − 3	2.26	2.54	1.90	4
2 − 4	0.86	1.06	1.32	1
3 − 4	2.23	1.92	3.68	6

The signed rank test in Tables 5–12 also shows that the improvement of separability is not consistent among classes. Only subject S6 showed a significant improvement for all pairwise classes between run ***D***_*i*_ relative to ***D***_1_. However, for other subjects, the change of separability is random across pair-wise classes and runs.

### Feature separability visualization

**Fig 6:**
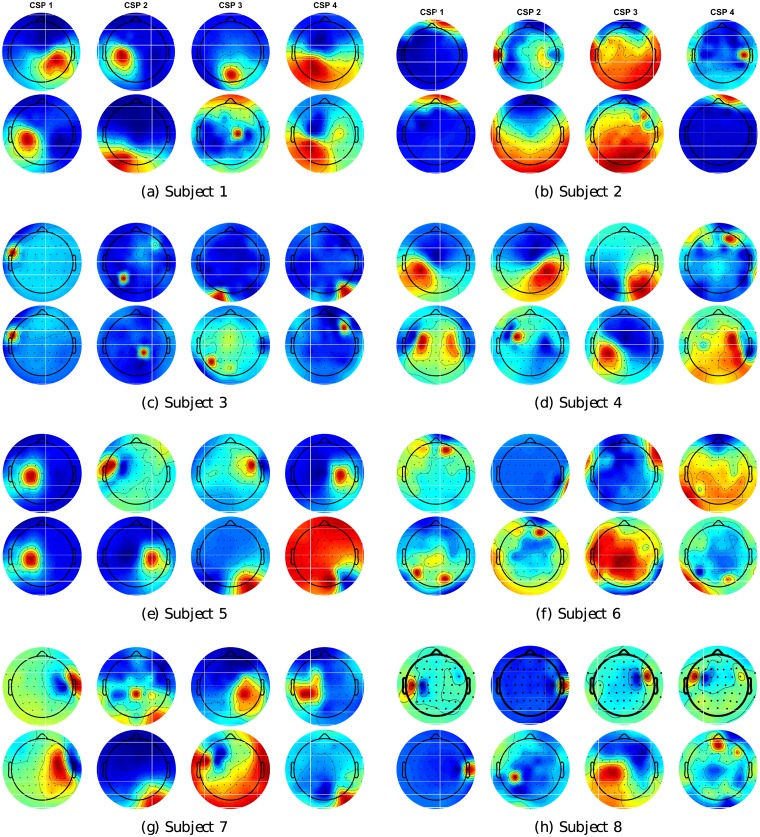
Multi-class Common Spatial Pattern topology. The first 4 CSP patterns extracted from Run 1 (first row) and the run thats has the highest averaged separability (second row) of 8 subjects.

Following the conventional methods, we analyze the difference of the CSP topology plot between the first run and the run with the highest separability for each subject. The multi-classes CSP method [48] forms the CSP matrix by first performing Independent Component Analysis and then ranking the components based on their Mutual Information scores with each mental task from highest to lowest. Hence, the first 4 components theoretically contain the most information about the classes and are selected to be shown in Fig 6.

However, the explanation for the CSP topology using this method is not as straight forward as the conventional binary CSP which has a few components. At best, we can observe the components at the middle and parietal sides of the brain, which lie over the motor cortex area (C3,C4,CZ) and Wernicke’s area, for Subject S1, S4, S5, S7 and S8. The ranking of components may not be consistent, as we can see that the component CSP2 in the first run reappears as CSP1 for Subject 1, or CSP 1 reappears as CSP 3 for subject 4, and CSP 4 reappears as CSP 2 for subject 5. Hence, this justifies the usage of a high number of CSP components, up to 12 in our method, to capture the most significant information at the preprocessing step.

While the CSP topology can help us understand the important channels, the Riemannian feature does not rely on each single CSP channel, but further captures the relationship between them. Hence, to better understand the data separability, we further visualize the distribution of the COV features.

Here, although we defined the separability using the Riemannian distance [51], visualizing the COV descriptor in the original Riemannian manifold is challenging. Therefore, we first map the COV descriptors into the Tangent vectors in Euclidean space, then map these highly dimensional vectors into 2D plane using the well-known method t-Distributed Stochastic Neighbor Embedding (t-SNE) [59]. We emphasize that mapping from the Riemannian space into the Euclidean space flattens the manifold and cannot fully preserve the distance between the features. However, we can then utilize well-established methods in Euclidean space for the visualization with acceptable accuracy.

**Fig 7:**
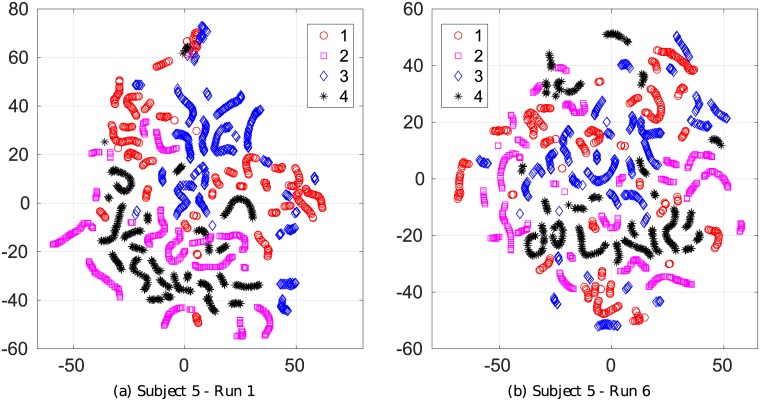
t-SNE visualization of tangent vector feature for run 1 and run 6 of subject 5.

Fig 7 shows the visualization of the tangent vector embedded in 2D space by the t-SNE algorithm for Subject 5 on run 1 and run 6. There are totally 330 features for each class, represented by markers of different colors and shapes in the figure. t-SNE is a nonlinear, unsupervised dimension reduction technique that can preserve as much as possible the relative distances between objects from the original space to the lower dimensional space. Our implementation used the built-in Matlab tSNE function, and set the hyper parameter perplexity equal to 20. Note that, although t-SNE is one of the best techniques currently, it can’t create a unique solution and still suffers from the intrinsic information loss of the embedding process. Nevertheless, it helps us gain some insights about the data distribution. As seen in Fig 7, features from the same trial are mapped close together into a small fragment.

Furthermore, class 1 (red) and class 3 (blue) are well separated from class 2 (purple) and class 4 (black), while the pair (1-3) and the pair (2-4) are not linearly separable. This matches with our previous claim of separability, that “the separability of the speech imagery pair (2-4) and the motor imagery pair (1-3) are the lowest, while the ones for the pair (“Left hand”-“Split”) (1-4) and (“Right Hand”,“Split”)(3-4) are the most discriminable.” However, it is not clear how to compare the level of separability between two runs using tSNE visualization, since the solutions are not unique and depend on selecting the hyper-parameter.

### User exploration and exploitation via data’s instability

The degree of user’s adaptation can be observed via the data’s instability of each class. Concretely, a high instability score corresponds to highly variant data, which indicates a high level of exploration. In reverse, a smaller one can be associated with low exploration, e.g, high level of exploitation. Here, we define the data’s instability for each class, not for the whole dataset. Thus, other factors that may affect the data variance, such as technical reasons, should lead to a consistent increasing or decreasing for all classes in a run. However, the data instability vary randomly across classes and runs. Since the user exploitation and exploration process for each class is the main contributor for class-wise variance, we can use this metric to quantify user adaptation level.

**Fig 8:**
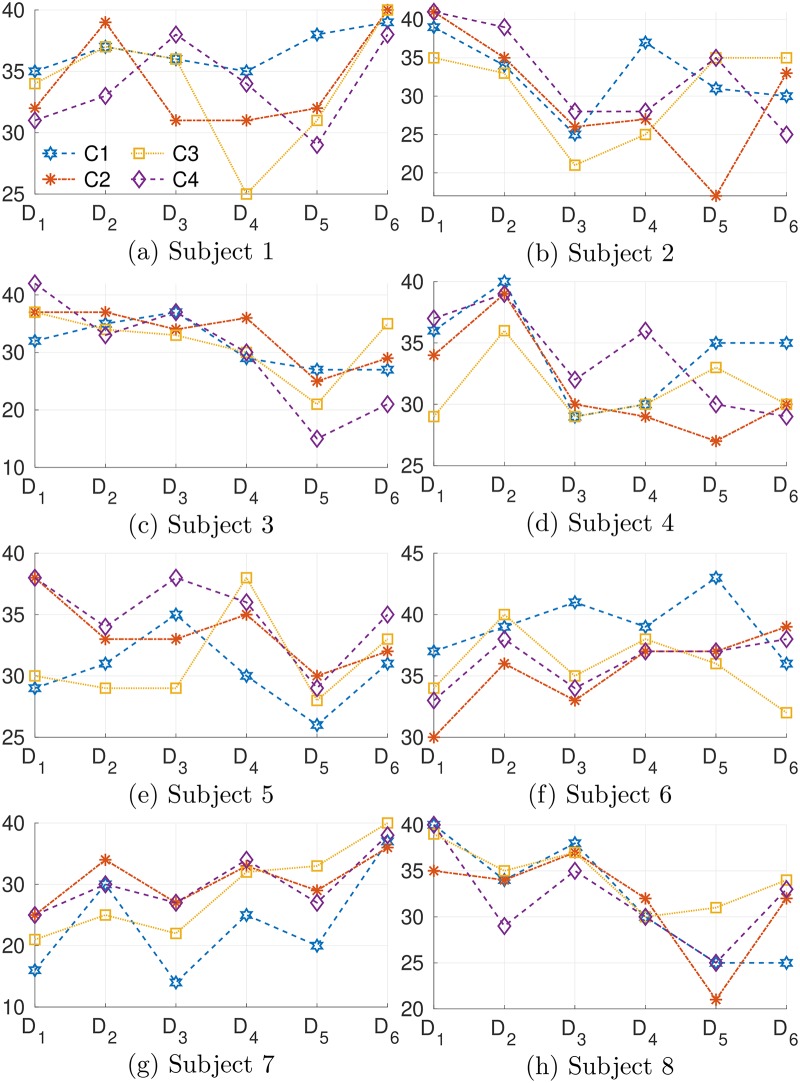
Data instability for each class along the runs. The legend notations of each class (C1-C4) are given in (a).

Fig 8 shows the evolution of the data instability through the runs. As we should expect, different users have different levels of adaptation for each class, depending on the feedback from the classifier. However, we can still observe the tendency of reducing instability from run 1 to run 4 or even run 5, such as in Subjects S2, S3, S4 and S8, which indicates that the users became more familiar to the systems and tried to apply what they had learned, e.g. exploitation. In run 6, we observe the increase of instability for subjects S1, S3, S6, S7 and S8. This indicates that the users felt unsatisfied with results in run 5, and explored new skills to deal with the change of feedback.

## Discussion

### Effectiveness of the mixture models

According to the experiment results, only Subject S6 showed improvement in both QCM and the data separability. Especially, subject S6 started near the chance level, i.e. 21.5% QCM, but achieved a consistent improvement up to 43% QCM. For this subject, the overall improvement can be contributed to both user and machine in the co-adaptation eco-system.

For other subjects, although the data separability varied randomly, e.g. increase in several pair-wise classes but decrease in the other, owning to the adaptive mixtures of classifier, the classification results still improved. For this group of subjects, the machine learning part is the main reason leading to the overall improvement. Note that, subject S8 also started near the chance level, i.e. 10.2% QCM, but achieved a consistent improvement up to 36% confusion matrix quality.

### How did the user learn to adapt to the BCI system

After the experiment, we had a short discussion with each participant and received very positive feedback. All of the subjects that participated in the previous offline BCI experiment reported that they were much more involved and concentrated in this experiment. For novice subjects with BCI, they shared a similar opinion that the experiment was actually quite fun, and more like playing a game.

For the question of how they performed imagination, some of their answers were: “At the beginning, I was not quite sure how to perform motor imagery. Later, I imagined that I grasped a ball, and I was changing the intensity when I grasped it. For speech imagery, sometimes I also imagined tearing a paper when I was saying “split”. For “concentration”, I adjusted the speed of saying the word.” (S7). “I imagined that I closed my hand and punched something when I performed motor imagery.” (S4). “I imagined how to pronounce the word and how it sounded in my head.” (S5). All the subjects admitted that the full-feedback was very challenging at the first time (run 5), but got used to it later (run 6).

### Comparison to literature

A comprehensive comparison with online BCI systems in literature is difficult since the approaches are very different in experimental protocols, and subject categories. In general, the majority of online BCI systems [5, 19–24, 26, 58] use binary classification, and aim to obtain classification accuracies above 70%, or equivalent with a Cohen’s *κ* = 0.4. An equal *κ* value for the case of 4 classes yields 55% accuracy. A grand average of the accuracy in our results is 52.5%, which is only slightly below the requirement. In addition, except subjects S3 and S8, all other subjects had accuracy scores significantly above the chance level. Nevertheless, as mentioned, our approach uses accuracy only as a reference metric.

For adaptive multi-class BCI systems, there are only a few previous works. Nicolas-Alonso et. al. [60] proposed an intricate algorithm, where the features are extracted from 9 Finite Impulse Response (FIR) bandpass filters, each of which is followed by a CSP filter. The most discriminant features are then selected based on mutual information by 10-fold cross validation training section. Each new feature vector is then centralized by subtracting for the mean of the training dataset. This mean vector is also re-estimated after every new sample using a forgetting factor. Finally, a semi-supervised Spectral Regression Kernel Discriminant Analysis is used to classify the feature. In their later work [61], the same procedure of extracting features is combined with a stacked classifier, in which the output from several regularized LDA (level 0) on different domains, such as spatial, spectral and temporal information, are stacked to the final classifier (level 1). Our system shares several characteristics with this approach, such as reestimating the feature mean and combining a set of classifiers. However, our approach updates the model after each run, and utilizes a mixture of models. This is because we aim to fix the Machine Learning part during the online test so that the human can explore and exploit techniques to adapt toward the system. Deliberating feature extraction for each subject such as in [60, 61] can potentially improve our proposed method.

The approach proposed by Spuler et. al. [15] adopts a new sample to retrain SVM if its prediction probability is greater than *p*_threshold_ = 0.8. A problem is that the selection then critically depends on the pre-trained classifier. For multiple-class prediction, if the pre-trained classifier is biased away a particular class, at the extreme, it will never predict a sample as this class. Consequently, no new sample of this class will be added to update the model, thus lead to imbalance of the training data and continue falsifying the bias. In contrast, our approach decouples the selection of new samples from the performance of the classifier so that, a new training dataset is always balanced, and can reflect what the user is exploring. Thus, a new RVM trained on this set can adapt toward the user tendency, rather than force the user to follow an initial, possibly inaccurate, pre-trained system.

The closest work to ours can be referred to the method proposed by Llera et. al. [62], in which the tangent vector of spatial covariance matrix is used as the feature, and the binary pooled mean LDA introduced in [22] is generalized for the multi-class case. Different from our approach, their method follows the sample-based adaptation, where the LDA’s mean is updated after every new sample in an unsupervised manner, e.g. for all data points. Our approach, in contrast, essentially follows the batch-based adaptation, where we update the geodesic mean reference point and RVM model every run. Moreover, not all but only representative data are selected to update the models.

## Conclusion

This paper proposed an adaptive, visual feedback based online BCI paradigm toward improving the efficiency of the conventional offline BCI. The framework successfully addresses our objectives. First, the system only needs a minimal time to calibrate, i.e. 8 minutes for 4 classes, and the users immediately receive feedback on how to use the system. Second, the mechanism of selecting representative data for updating the models and the adaptive mixture of RVM models results in the improvement of classification performance, while encouraging participants to explore and exploit their mental processes in their own way. Third, we combined different modalities of mental tasks, namely motor imagery and speech imagery, to increase the DoF for BCI applications. All participants demonstrated significant improvements based on the confusion matrix quality criterion. Data separability is used to evaluate the user performance separately, and demonstrate the effectiveness of the co-adaptive system. Furthermore, the instability of data is used as the indication of the exploration and exploitation learning process. We received very positive feedback from users, which once again emphasizes the importance of early feedback on BCI applications. The proposed method can be improved further by refining spatial filter and incorporating features from the frequency domain. A future work can also be extending to a higher number of DoF, and subject- dependent features.

## References

[1] Tanaka K, Matsunaga K, Wang HO. Electroencephalogram-based control of an electric wheelchair. IEEE Trans Robot. 2005;21(4):762–766. 10.1109/TRO.2004.842350

[2] Neuper C, Klimesch W. Event-related dynamics of brain oscillations. Elsevier; 2006.

[3] Guger C, Edlinger G, Harkam W, Niedermayer I, Pfurtscheller G. How many people are able to operate an EEG-based brain-computer interface (BCI)? IEEE Trans Neural Syst Rehabil Eng. 2003;11(2):145–147. 10.1109/TNSRE.2003.814481 12899258

[4] Kübler A, Neumann N, Wilhelm B, Hinterberger T, Birbaumer N. Predictability of Brain-Computer Communication. J Psychophysiol. 2004;18(2/3):121–129. 10.1027/0269-8803.18.23.121

[5] Vidaurre C, Sannelli C, Müller KR, Blankertz B. Machine-learning-based coadaptive calibration for brain-computer interfaces. Neural computation. 2011;23(3):791–816. 10.1162/NECO_a_00089 21162666

[6] Brunner C, Leeb R, Muller-Putz G R, Schlogl A, Pfurtscheller G BCI Competition 2008—Graz data set A. Institute for Knowledge Discovery, and Institute for Human-Computer Interfaces Graz University of Technology, Austria; 2008.

[7] Wester M, Schultz T. Unspoken speech-speech recognition based on electroencephalography. Universität Karlsruhe (TH),Karlsruhe, Germany; 2006.

[8] D’Zmura M, Deng S, Lappas T, Thorpe S, Srinivasan R. Toward EEG Sensing of Imagined Speech. Int Conf Human-Computer Interact. 2009; p. 40–48.

[9] Denby B, Schultz T, Honda K, Hueber T, Gilbert JM, Brumberg JS. Silent speech interfaces. Speech Commun. 2010;52(4):270–287. 10.1016/j.specom.2010.02.001

[10] Kim J, Lee SK, Lee B. EEG classification in a single-trial basis for vowel speech perception using multivariate empirical mode decomposition. J Neural Eng. 2014;11(3):036010 10.1088/1741-2560/11/3/036010 24809722

[11] Brigham K, Kumar BVKV. Imagined speech classification with EEG signals for silent communication: A preliminary investigation into synthetic telepathy. 2010 4th Int Conf Bioinforma Biomed Eng iCBBE 2010. 2010; p. 1–4.

[12] DaSalla CS, Kambara H, Sato M, Koike Y. Single-trial classification of vowel speech imagery using common spatial patterns. Neural Networks. 2009;22(9):1334–1339. 10.1016/j.neunet.2009.05.008 19497710

[13] Deng S, Srinivasan R, Lappas T, D’Zmura M. EEG classification of imagined syllable rhythm using Hilbert spectrum methods. J Neural Eng. 2010;7(4):046006 10.1088/1741-2560/7/4/046006 20551510

[14] Wang L, Zhang X, Zhong X, Zhang Y. Analysis and classification of speech imagery EEG for BCI. Biomed Signal Process Control. 2013;8(6):901–908. 10.1016/j.bspc.2013.07.011

[15] Spüler M, Rosenstiel W, Bogdan M. Adaptive SVM-based classification increases performance of a MEG-based Brain-Computer Interface (BCI). Lecture Notes in Computer Science (including subseries Lecture Notes in Artificial Intelligence and Lecture Notes in Bioinformatics). 2012;7552 LNCS(PART 1):669–676.

[16] Diehl CP, Cauwenberghs G. Svm incremental learning, adaptation and optimization. Proceedings of the International Joint Conference on Neural Networks, 2003;4:2685–2690.

[17] Spüler M, Rosenstiel W, Bogdan M. Principal component based covariate shift adaption to reducenon-stationarity in a MEG-based brain-computer interface. EURASIP Journal on Advances in Signal Processing. 2012;2012(1):129 10.1186/1687-6180-2012-129

[18] Vidaurre C, Schlögl A, Cabeza R, Scherer R, Pfurtscheller G. Adaptive Online Classification for EEG-based Brain Computer Interfaces with AAR parameters and band power estimates. Biomed Tech Eng. 2005;50(11):350–354. 10.1515/BMT.2005.049 16370147

[19] Vidaurre C, Cabeza R, Scherer R, Pfurtscheller G. Fully On-Line Adaptive BCI. IEEE Trans Biomed Eng. 2005;10870(6):1049–1050. 10.1109/TBME.2006.873542 16761852

[20] Vidaurre C, Cabeza R, Scherer R, Pfurtscheller G. Study of On-Line Adaptive Discriminant Analysis for EEG-Based Brain Computer Interfaces. IEEE Trans Biomed Eng. 2007;54(3):550–556. 10.1109/TBME.2006.888836 17355071

[21] Vidaurre C, Sannelli C, Müller KR, Blankertz B. Co-adaptive calibration to improve BCI efficiency. J Neural Eng. 2011;8(2). 10.1088/1741-2560/8/2/025009 21436515

[22] Vidaurre C, Kawanabe M, Von Bünau P, Blankertz B, Müller KR. Toward unsupervised adaptation of LDA for brain-computer interfaces. IEEE Trans Biomed Eng. 2011;58(3 PART 1):587–597. 10.1109/TBME.2010.2093133 21095857

[23] Faller J, Vidaurre C, Solis-Escalante T, Neuper C, Scherer R. Autocalibration and recurrent adaptation: Towards a plug and play online ERD-BCI. IEEE Trans Neural Syst Rehabil Eng. 2012;20(3):313–319. 10.1109/TNSRE.2012.2189584 22481835

[24] Faller J, Scherer R, Costa U, Opisso E, Medina J, Müller-Putz GR. A co-adaptive brain-computer interface for end users with severe motor impairment. PLoS One. 2014;9(7):1–10. 10.1371/journal.pone.0101168 PMC4094431 25014055

[25] Scherer R, Faller J, Opisso E, Costa U, Steyrl D, Muller-Putz GR. Bring mental activity into action! An enhanced online co-adaptive brain-computer interface training protocol. Proc Annu Int Conf IEEE Eng Med Biol Soc EMBS. 2015;2015-Novem:2323–2326. 10.1109/EMBC.2015.7318858 26736758

[26] Faller J, Solis-Escalante T, Costa U, Opisso E, Medina J, Scherer R, et al Online co-adaptive brain-computer interfacing: Preliminary results in individuals with spinal cord injury. Int IEEE/EMBS Conf Neural Eng NER. 2013;3:977–980.

[27] Merel J, Pianto DM, Cunningham JP, Paninski L. Encoder-Decoder Optimization for Brain-Computer Interfaces. PLoS Computational Biology. 2015;11(6):1–25. 10.1371/journal.pcbi.1004288 PMC4451011 26029919

[28] Lotte F, Jeunet C, Lotte F, Jeunet C. Online classification accuracy is a poor metric to study mental imagery-based bci user learning: an experimental demonstration and new metrics. In: 7th Inter- Natl. BCI Conf.; 2017.

[29] Müller JS, Vidaurre C, Schreuder M, Meinecke FC, von Bünau P, Müller KR. A mathematical model for the two-learners problem. Journal of Neural Engineering. 2017;14(3):036005 10.1088/1741-2552/aa620b 28224972

[30] Nguyen CH, Karavas G, Artemiadis P. Inferring imagined speech using EEG signals: a new approach using Riemannian Manifold features. J Neural Eng. 2017. 10.1088/1741-2552/aa8235 28745299

[31] Porikli F, Tuzel O, Meer P. Covariance tracking using model update based on Lie algebra. Proc IEEE Comput Soc Conf Comput Vis Pattern Recognit. 2006;1(January):728–735.

[32] Tuzel O, Porikli F, Meer P. Human Detection via Classification on Riemannian Manifolds. Comput Vis Pattern Recognition, 2007 CVPR’07 IEEE Conf. 2007; p. 1–8. 10.1109/TPAMI.2008.75 18703826

[33] Lui YM. Human gesture recognition on product manifolds. Jmlr. 2012;13:3297–3321.

[34] Barachant A, Bonnet S, Congedo M, Jutten C. Common Spatial Pattern revisited by Riemannian geometry. Multimed Signal Process (MMSP), 2010 IEEE Int Work. 2010; p. 472–476. 10.1109/MMSP.2010.5662067

[35] Barachant A, Bonnet S, Congedo M, Jutten C. Multiclass brain-computer interface classification by Riemannian geometry. IEEE Trans Biomed Eng. 2012;59(4):920–928. 10.1109/TBME.2011.2172210 22010143

[36] Barachant A, Bonnet S, Congedo M, Jutten C. Classification of covariance matrices using a Riemannian-based kernel for BCI applications. Neurocomputing. 2013;112:172–178. 10.1016/j.neucom.2012.12.039

[37] Congedo M, Barachant A, Andreev A. A New Generation of Brain-Computer Interface Based on Riemannian Geometry. arXiv Prepr arXiv13108115. 2013;33(0).

[38] Samek W, Kawanabe M, Muller KR. Divergence-based framework for common spatial patterns algorithms. IEEE Rev Biomed Eng. 2014;7:50–72. 10.1109/RBME.2013.2290621 24240027

[39] Yger F, Berar M, Lotte F. Riemannian approaches in Brain-Computer Interfaces: a review. IEEE Trans Neural Syst Rehabil Eng. 2016;4320(c):1–10. 10.1109/TNSRE.2016.2627016 27845666

[40] Wang L, Zhang X, Zhang Y. Extending motor imagery by speech imagery for brain-computer interface. In: 2013 35th Annual International Conference of the IEEE Engineering in Medicine and Biology Society (EMBC); 2013. p. 7056–7059. 10.1109/EMBC.2013.6611183 24111370

[41] Karavas G, Larsson D, Artemiadis P. A hybrid brain-machine interface for control of robotic swarms: Preliminary results. IEEE/RSJ Int Conf Intell Robot Syst. 2017.

[42] Klem G, Luders H, Jasper H, Elger C. The ten-twenty electrode system of the International Federation. Electroencephalogr Clin Neurophysiol. 1958;10(2):371–375. 10590970

[43] He P, Wilson G, Russell C. Removal of ocular artifacts from electro-encephalogram by adaptive filtering. Med Biol Eng Comput. 2004;42(3):407–412. 10.1007/BF02344717 15191087

[44] Koles ZJ, Lazar MS, Zhou SZ. Spatial patterns underlying population differences in the background EEG. Brain Topogr. 1990;2(4):275–284. 10.1007/BF01129656 2223384

[45] Dornhege G, Blankertz B, Curio G, Muller KR. Increase information transfer rates in BCI by CSP extension to multi-class. Adv Neural Inf Process Syst. 2004;16:733–40.

[46] Wang Yujun, Sangkai Gao XG. Common Spatial Pattern Method for Channel Selection in Motor Imagery Based Brain-Computer Interface. Eng Med Biol. 2005;5:5392–5395. 10.1109/IEMBS.2005.1615701 17281471

[47] Blankertz B, Tomioka R, Lemm S, Kawanabe M, Müller KR. Optimizing Spatial Filters for Robust EEG Single-Trial Analysis. IEEE Signal Process Mag. 2008;XX:1–12.

[48] Grosse-Wentrup M, Buss M. Multiclass Common Spatial Patterns and Information Theoretic Feature Extraction. IEEE Trans Biomed Eng. 2008;55(8):1991–2000. 10.1109/TBME.2008.921154 18632362

[49] Tu LW. An introduction to manifolds. Springer Science & Business Media; 2010.

[50] Nguyen CH, Artemiadis P. EEG Feature Descriptors and Discriminant Analysis under Riemannian Manifold perspective. Neurocomputing. 2017.

[51] Pennec X, Fillard P, Ayache N. A Riemannian Framework for Tensor Computing. Int J Comput Vis. 2006;66(5255):41–66. 10.1007/s11263-005-3222-z

[52] Karcher H. Riemannian center of mass and mollifier smoothing. Commun Pure Appl Math. 1977;30(5):509–541. 10.1002/cpa.3160300502

[53] Moakher M. A Differential Geometric Approach to the Geometric Mean of Symmetric Positive-Definite Matrices. SIAM J Matrix Anal Appl. 2005;26(3):735–747. 10.1137/S0895479803436937

[54] Fiori S, Tanaka T. An algorithm to compute averages on matrix lie groups. IEEE Trans Signal Process. 2009;57(12):4734–4743. 10.1109/TSP.2009.2027754

[55] Tipping M. Sparse Bayesian Learning and the Relevance Vector Mach. J Mach Learn Res. 2001;1:211–244.

[56] Damoulas T, Girolami MA. Probabilistic multi-class multi-kernel learning: On protein fold recognition and remote homology detection. Bioinformatics. 2008;24(10):1264–1270. 10.1093/bioinformatics/btn112 18378524

[57] Psorakis I, Damoulas T, Girolami MA. Multiclass Relevance Vector Machines: Sparsity and Accuracy. IEEE Trans Neural Networks. 2010; p. 1–11. 10.1109/TNN.2010.2064787 20805053

[58] Faller J, Scherer R, Friedrich EVC, Costa U, Opisso E, Medina J, et al Non-motor tasks improve adaptive brain-computer interface performance in users with severe motor impairment. Front Neurosci. 2014;8(OCT):1–11. 25368546 10.3389/fnins.2014.00320 PMC4196541

[59] Van Der Maaten LJP, Hinton GE. Visualizing high-dimensional data using t-sne. Journal of Machine Learning Research. 2008;9:2579–2605.

[60] Nicolas-Alonso LF, Corralejo R, Gomez-Pilar J, Álvarez D, Hornero R. Adaptive semi-supervised classification to reduce intersession non-stationarity in multiclass motor imagery-based brain-computer interfaces. Neurocomputing. 2015;159(1):186–196. 10.1016/j.neucom.2015.02.005

[61] Nicolas-Alonso LF, Corralejo R, Gomez-Pilar J, Álvarez D, Hornero R. Adaptive Stacked Generalization for Multiclass Motor Imagery-Based Brain Computer Interfaces. IEEE Trans Neural Syst Rehabil Eng. 2015;23(4):702–712. 10.1109/TNSRE.2015.2398573 25680208

[62] Llera A, Gómez V, Kappen HJ. Adaptive multiclass classification for brain computer interfaces. Neural Comput. 2014;26(6):1–19. 10.1162/NECO_a_00592 24684452

